# Early skin colonization by *Staphylococcus epidermidis* and *Staphylococcus aureus* reveals environment-dependent synergistic effects

**DOI:** 10.3389/fmicb.2025.1677214

**Published:** 2025-10-02

**Authors:** Chrisse Ngari, Valérie Poulet, Giuseppe Percoco, Laurent Peno-Mazzarino, Damien Seyer

**Affiliations:** ^1^Laboratoires Clarins, Pontoise, France; ^2^Eurofins BIO-EC, Longjumeau, France; ^3^ERRMECe Laboratory, CY Cergy-Paris Université, Cergy-Pontoise, France

**Keywords:** skin microbiome, reconstructed human epidermis, skin explant, *Staphylococcus epidermidis*, *Staphylococcus aureus*, barrier function

## Abstract

**Introduction:**

The skin microbiome is crucial for skin health and homeostasis. It contributes to immune defense, promotes epidermal differentiation, and supports a robust skin barrier function. Despite its importance, few studies have used model skin systems to examine how simple bacterial communities interact and how they impact the skin.

**Methods:**

We studied the interaction between a skin commensal, *Staphylococcus epidermidis*, and a pathogen, *Staphylococcus aureus*, by performing inoculations and co-inoculations on Reconstructed Human Epidermis (RHE) models maintained under classical humid conditions (>90% relative humidity) or dry conditions (<25% RH). In parallel, inoculations were conducted on human skin explants using a novel culture setup preserving physiological humidity levels (40–60% RH). Bacterial attachment was assessed 4 h post-inoculation. At 24 h, histology was examined, three natural moisturizing factors (NMFs) were quantified. Filaggrin (FLG) and ceramide levels were analyzed to assess the skin barrier function.

**Results:**

In contrast to previous findings at 24 h, co-inoculation increased *S. epidermidis* and *S. aureus* attachment in RHEs, but only under humid conditions. Only RHE maintained in dry conditions and skin explant revealed an effect of co-inoculation on filaggrin (FLG), yet an increase in RHEs and a decrease in skin explants. In both RHEs maintained in dry conditions and skin explants, NMF levels were consistently reduced following co-inoculation. In RHEs and skin explants, inoculation with *S. aureus* alone also lowered NMFs, with co-inoculation further amplifying this effect. Finally, ceramide levels increased similarly across both inoculations and co-inoculation.

**Discussion:**

Revealing unexpected early interactions between *S. epidermidis*, *S. aureus*, and the skin, our results could suggest that co-inoculation may trigger a synergetic disruption of the barrier function. Alternatively, it could imply that co-inoculation might reinforce the epidermal barrier via a *S. aureus*-mediated stimulation of the protective functions of *S. epidermidis*. Further studies are needed to confirm these effects and determine whether they are strain-specific or more broadly applicable.

## 1 Introduction

As a continuous organ covering the entire body, the skin serves as a critical barrier between the internal and external environments. This barrier function primarily relies on the stratum corneum (SC), the outermost skin layer, which fulfills physical, chemical, and microbial protective functions (Lefèvre-Utile et al., 2021; de Szalay and Wertz, 2023). The distinctive structure and composition of the SC, consisting of terminally differentiated keratinocytes – the corneocytes – embedded in a highly organized lipidic lamellar matrix, largely account for this protective mechanism (Elias, 1983; Baroni et al., 2012).

Filaggrin (FLG), a major constituent of corneocytes, is pivotal to the barrier function (Drislane and Irvine, 2020). Initially present as pro-FLG, enzymatic processing leads to FLG that aggregates with keratin filaments (Briot et al., 2020; Quiroz et al., 2020). This network binds to the keratinocyte membrane, resulting in its collapse and the flattened corneocyte shape that contributes to the physical strength of the external skin layer. As maturation proceeds, FLG undergoes fragmentation into hygroscopic amino acids and their metabolites, the natural moisturizing factors (NMFs), which are essential for SC hydration (Rawlings and Harding, 2004). Proper hydration ensures corneocyte maturation, desquamation, and SC homeostasis (Rawlings and Harding, 2004). It is under the tight control of internal factors and external relative humidity, both of which regulate FLG degradation into NMFs (Cau et al., 2017). Skin hydration is also critical for SC flexibility and contributes to skin suppleness. It also has important cosmetic properties, dry skin being rough, scaly, flaky, and itchy.

In addition to these endogenous mechanisms, the skin microbiome, comprising bacteria, fungi, viruses, and microeukaryotes, has emerged as a key player in skin health and homeostasis (Flowers and Grice, 2020). It is involved in skin defense and immune responses. Bacteria such as *Staphylococcus epidermidis*, one of the most common skin commensals, can produce toxins that, along with antimicrobial skin peptides, selectively kill pathogens such as *Staphylococcus aureus* (Cogen et al., 2010a, 2010b). The microbiome also stimulates genes from the epidermal differentiation complex, promoting epidermal differentiation and participating in the skin barrier function (Meisel et al., 2018). Furthermore, *S. epidermidis* increases the skin’s lipid content, thereby limiting water evaporation and improving skin hydration (Nodake et al., 2015). *S. epidermidis* also secretes lipases that hydrolyze sebum and can ferment glycerol. Both processes produce organic fatty acids, especially lactic acid, which help maintain acidic conditions (Nodake et al., 2015). These acidic conditions contribute to the inhibition of pathogenic bacteria, and lactic acid helps improve skin hydration. Conversely, a bacterium such as *S. aureus* downregulates FLG expression, thereby affecting skin hydration. Moreover, FLG deficiency favors *S. aureus* colonization, leading to a vicious cycle (Kim and Lim, 2021).

While numerous studies have evaluated the diversity of the skin microbiome, very few have investigated inter-species interactions. An early work used immobilized keratinocytes and simplified commensal-pathogen community models to demonstrate that commensals restrict pathogen growth and biofilm formation, thus limiting damages to keratinocytes (Jordana-Lluch et al., 2020). Although reconstructed human epidermis (RHE) models reflect the skin and its epidermal barrier, only a few studies have analyzed single-species colonization (Rademacher et al., 2018; Ochlich et al., 2023). A single work took advantage of RHE to investigate a commensal-pathogen community (Kohda et al., 2021), showing that *S. epidermidis* restricts *S. aureus* colonization, limits *S. aureus*-induced cytotoxicity and secretion of proinflammatory cytokines, also preventing *S. aureus* from entering deep cell layers.

While RHE is a model of choice, it suffers some limitations. In particular, the SC present an altered lipid composition and organization, resulting in a weakened barrier function (Thakoersing et al., 2012). While skin explants do not suffer these limitations, there are maintained under a minimum of 90% relative humidity and 5% carbon dioxide, which are far from physiological conditions. In addition, they are maintained entirely at 37 °C, not presenting the 32–37 °C temperature gradient that exist *in vivo* and that is important for a normal hydration of the SC as well as for the proper proliferation and differentiation of keratinocytes (Spencer et al., 1975; Borowiec et al., 2013). To circumvent these limitations, a new culture system, the *Perfex* setup, was recently developed, enabling to maintain skin explants in conditions very close to physiological conditions (Peno-Mazzarino et al., 2024).

Thus, to investigate the interaction between *S. epidermidis*, *S. aureus*, and the skin, we used RHEs that were maintained under the “classical” humid conditions (37 °C, 5% CO_2_, >90% relative humidity), also assessing the effects of dry conditions closer to “normal” conditions (37 °C, 5% CO_2_, <25% relative humidity). We also took advantage of the new *Perfex* setup that enable maintaining skin explants under physiological conditions (32 °C, 0.04% CO_2_ and 40–60% relative humidity). In all these models, we evaluated bacteria attachment and analyzed hydration and barrier function-related markers in RHEs upon *S. epidermidis* or *S. aureus* inoculation and *S. epidermidis* - *S. aureus* co-inoculation.

## 2 Materials and methods

### 2.1 Bacterial strains and growth conditions

*Staphylococcus epidermidis* and *Staphylococcus aureus* strains producing easily distinguishable colonies in terms of size and color, were selected. *S. epidermidis* was either the strain Fussel 14990™ (RHE experiments) or CIP 107777 (*Perfex* skin explant experiments). Despite some differences, both strains are classical model strains of *S. epidermidis* commensals. For the inoculation of both RHEs and skin explants, the *Staphylococcus aureus* subsp. *aureus* ATCC 6538™ was used. All strains were stored at −80 °C and subcultured at 37 °C on Tryptone Soy Agar plates or in Tryptone Soy Broth.

### 2.2 Reconstituted human epidermis (RHE)

For RHEs, primary human keratinocytes from an adult donor were cultured on 0.6 cm^2^ porous polycarbonate inserts (Millipore, #PIHP01250) before being exposed to the air-liquid interface under either classical humid conditions (37 °C, 5% CO_2_, >90% relative humidity) or dry conditions (37 °C, 5% CO_2_, <25% relative humidity). These humid or dry conditions were maintained thorough the experiment.

RHE were cultured for 8 d in optimized EpiLife® culture medium (Gibco, #MEPI500CA), supplemented with the Human Keratinocyte Growth Supplement kit (Gibco, #S0015) and penicillin/streptomycin (Sigma, #P433). The medium was renewed daily and, on day 8, replaced with antibiotic- and hydrocortisone-free EpiLife® medium. After 24 h (day 9), RHEs were inoculated and used for experiments.

Inoculation and subsequent analysis were conducted on three different RHEs from the same batch per condition and time point.

### 2.3 *Perfex* skin explants

A skin explant was obtained from an elective abdominoplasty performed on a 28-year-old Caucasian woman. Its procurement strictly complied Articles L.1245–2 and L.1211–1 of the French Public Health Code. Written informed consent was obtained from the donor.

After removal of the hypodermis, explant fragments were maintained using the *Perfex* setup (Peno-Mazzarino et al., 2024). Briefly, the dermal side was perfused with buffered, temperature-controlled BEM medium (BIO-EC’s Explant Medium), while the epidermal surface was exposed to “normal” conditions (32 °C, 0.04% CO_2_ and 40–60% relative humidity). Explants were inoculated after 3 d and used for experiments.

Inoculations were conducted on two different skin explants from the same donor per condition and time point, each being divided in three to conduct subsequent analysis in triplicates.

### 2.4 Bacterial inoculation and incubation

Overnight liquid bacterial cultures, in early stationary phases, were washed and resuspended in PBS buffer. For mono-inoculations, 1.5×10^5^ CFU/mm^2^ were deposited onto the surface of RHEs or skin explants. In the case of the strain mixture, both strains were applied at a 1:1 ratio, each at 1.5×10^5^ CFU/mm^2^.

*S. epidermidis* strain Fussel (14990™) and/or *S. aureus* subsp. *aureus* Rosenbach were used to inoculate RHEs. *S. epidermidis* CIP 4.83 and/or *S. aureus* strain ATCC 10577 were used to inoculate *Perfex* skin explants. Inoculum size was confirmed by plate count. Application of PBS buffer alone served as a negative control.

Inoculated RHE were incubated in dry (37 °C, 5% CO_2_, <25% relative humidity) or humid (37 °C, 5% CO_2_, >90% relative humidity) conditions. *Perfex* skin explants were incubated under “normal” conditions (32 °C, 0.04% CO_2_ and 40–60% relative humidity). The incubation was stopped after four and 24 h. Prior to further analysis, non-adherent bacteria were removed by four consecutive PBS buffer washes.

### 2.5 Bacterial numeration

RHEs were homogenized using the Percellys® system (Bertin Technologies). The surface of skin explants was gently scraped with a scalpel blade while washed with PBS. Serial dilutions of RHE homogenates and skin surface washings were plated on Tryptone Soy Agar. Colony-forming units (CFUs) were numerated after overnight incubation at 37 °C. *S. epidermidis* and *S. aureus* colonies were identified based on the very different colony morphology of the strains used, confirmed, in part of the experiments, using CHROMagar™ *S. aureus*.

Numeration results were obtained from a single bacterial enumeration performed 4 h after inoculation on three independent RHEs or on three fragments of the same skin explant 4 h after inoculation.

### 2.6 Histological analysis of RHE

Twenty-four hours after inoculation, RHEs and skin explants were fixed in formaldehyde, dehydrated in ethanol, and embedded in paraffin. Five-micrometer-thick cross-sections were stained with hematoxylin and eosin (RHEs) or with Masson’s trichrome staining (skin explants). Histology was evaluated using the Olympus SLIDEVIEW™ VS200 slide scanner and analyzed with the OlyVIA software (Olympus). Alternatively, a Leica DMLB or an Olympus BX63 microscope equipped with a DP Olympus digital camera were used. Analysis was conducted using the CellSens software (Olympus). A minimum of three representative images per replicate were assessed.

### 2.7 Analysis of NMFs

Among the various NMFs derived from FLG degradation, analyses focused on serine, pyrrolidone carboxylic acid (PCA), and urocanic acid. Analytes were extracted from RHEs using an aqueous extraction.

NMFs were quantified using an UltiMate™ 3,000 liquid chromatography system (Thermo Scientific, CA, USA) coupled with an ISQ EM mass detector (Thermo Scientific, CA, USA). Mobile phases [M1: water + 0.1% formic acid] and [M2: methanol + 0.1% formic acid] were eluted at a 0.4 mL/min flow rate. The injection volume was 20 μL, and the column temperature was maintained at 40 °C. For MS detection, Positive Electrospray Ionization (ESI+) was used. Data were analyzed using the Chromeleon™ 7.2 software (Thermo Fisher Scientific, MA, USA).

Results were obtained from measurements (conducted 24 h after bacterial inoculation) on three independent RHEs (each with duplicate technical repeats) or on three fragments of the same skin explant (each with triplicate technical repeats).

### 2.8 Analysis of pro-filaggrin and filaggrin proteins from RHEs

RHE or skin explant in RIPA buffer (Sigma, #R0278) supplemented with a protease inhibitor cocktail (Roche, #5892791001) were homogenized using the Precellys® system (Bertin Technologies). Following centrifugation, the supernatants were analyzed using the Wes™ capillary electrophoresis system (ProteinSimple). Proteins were separated by molecular weight, immobilized via UV, and total protein quantified using a specific dye. Filaggrin (FLG) monomers (50 kDa) were detected using a mouse anti-FLG primary antibody (Santa Cruz, #sc-66192) and a goat anti-mouse peroxidase-conjugated secondary antibody (ProteinSimple, #DM-002).

Quantifications were performed 24 h after bacterial inoculation on three independent RHEs, with each measurement carried out in duplicate technical repeats.

### 2.9 Immunohistochemical analysis of filaggrin, loricrin, and ceramides from skin explants

Formalin-fixed, paraffin-embedded skin explant sections were used for immunostaining. FLG was detected with a mouse monoclonal primary antibody (Santa Cruz, #sc-66192) and an Alexa Fluor™ 488-conjugated goat anti-mouse secondary antibody (Invitrogen, #A-11001). Ceramides were detected using a monoclonal anti-ceramide antibody (Glycobiotech, #MAB_0013). Detection relied on a biotin/streptavidin amplification system, revealed with the Vector® VIP peroxidase substrate (Vector Laboratories, #SK-4600). After image acquisition with an Olympus BX63 microscope equipped with an Olympus DP camera, signal intensity was quantified using the CellSens software (Olympus).

Immunohistochemical labeling was quantified from at least three representative fields in cross-sections of three fragments of the same skin explant.

### 2.10 Statistical analysis

All results are expressed as mean ± standard deviation. Normal data distribution was assessed using the Shapiro–Wilk tests (*α* = 0.01). Statistical comparisons were performed using Student’s *t*-tests or one-way ANOVA, followed by pairwise Tukey HSD tests. *p*-values below 0.05 were considered statistically significant, and those between 0.1 and 0.5 at the significance limit.

## 3 Results

### 3.1 Colonization of RHEs and skin explants by bacteria does not affect their morphology

**Figure 1:**
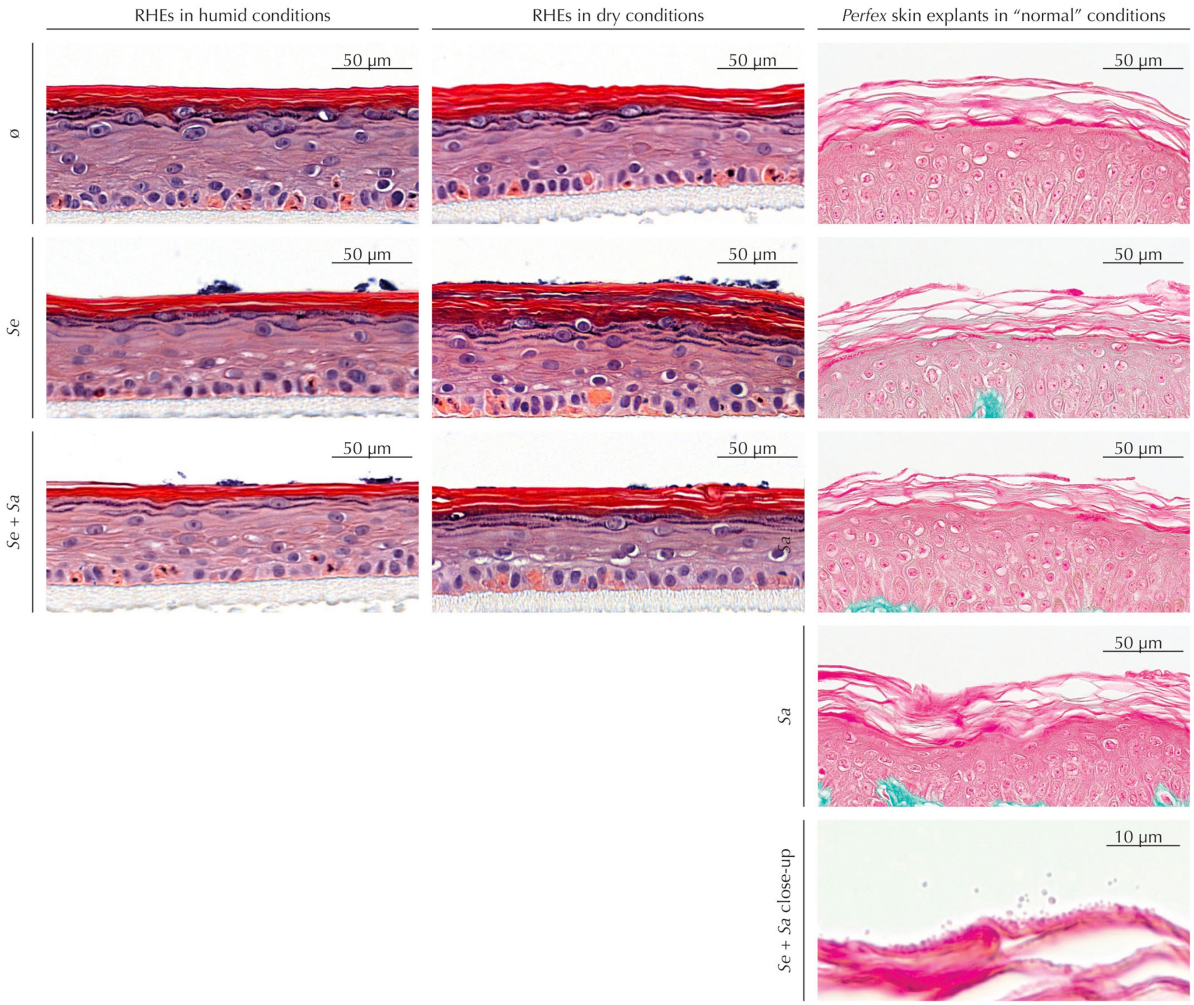
Histological staining of RHEs (hematoxylin and eosin staining) and *Perfex* skin explants (Masson’s trichrome staining) uninoculated or inoculated by *S. epidermidis* and/or *S. aureus*.

As a preliminary step, we used histological staining to ascertain that bacteria colonization had no observable impact on RHEs or skin explants morphology (Figure 1).

Uninoculated RHEs maintained in dry conditions exhibited the expected SC increased thickness compared to those subjected to humid conditions. This thickening was also observed in inoculated RHEs – either by *S. epidermidis* alone or by the *S. epidermidis* and *S. aureus* mixture. Regardless of humidity, colonization did not induce any histological alterations, although bacterial colonies were visible on the SC surface.

Similarly, skin explants showed no histological differences between inoculated and uninoculated samples, whatever the inoculum. While Masson’s trichrome staining provided limited visualization of bacteria at low magnification, higher magnifications revealed bacterial presence in the outermost SC layers.

### 3.2 Adhesion of bacteria

Bacterial adhesion was assessed by quantifying attached bacteria 4 h post-inoculation.

**Figure 2:**
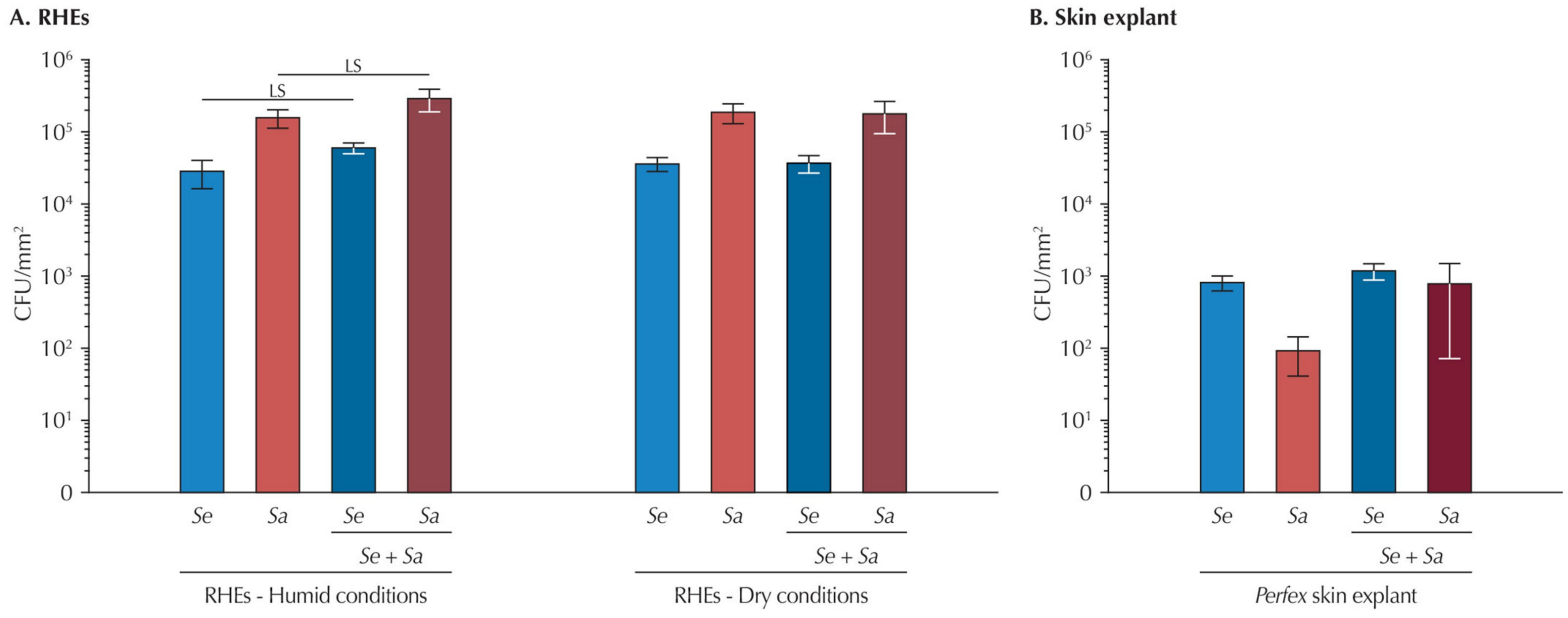
Bacteria attachment 4 h after inoculation **(A)** to RHEs and **(B)** to *Perfex* skin explants. Results are expressed as mean ± standard deviation from a single bacterial count performed on three different RHEs or on three fragments of the same skin explant. Statistical differences are reported with LS: *p* < 0.10 and <0.05.

On RHEs (Figure 2A), humid or dry conditions had no effect on the adhesion efficiency of either *S. epidermidis* or *S. aureus* when the two strains were inoculated individually (*p* = 0.567 and *p* = 0.634, respectively). However, *S. aureus* consistently adhered more effectively than *S. epidermidis* (9.3% of the *S. aureus* from the inoculum adhered to the RHE *versus* 2.1% of *S. epidermidis*, *p* = 0.037 in humid conditions; 11.13% *versus* 2.7%, respectively, *p* = 0.044 in dry conditions). Under humid conditions, co-inoculation modestly increased adhesion for both species compared to single-species inoculation (*S. epidermidis*: +38%; *S. aureus*: +39%) and the increase is at the significance limit (*S. epidermidis*: *p* = 0.088; *S. aureus*: *p* = 0.066). while a similar trend seems to exist in dry conditions, differences are not significant (*S. epidermidis*: *p* = 0.739; *S. aureus*: *p* = 0.901).

In skin explants (Figure 2B), adhesion efficiency at 4 h was approximately 100-fold lower than in RHEs (0.039% for *S. epidermidis* and 0.003% for *S. aureus*), with, contrary to what was observed on RHEs, *S. epidermidis* exhibiting better attachment. No differences were detected between single and co-inoculations for either bacterium.

### 3.3 Quantification of natural moisturizing factors (NMFs)

**Figure 3:**
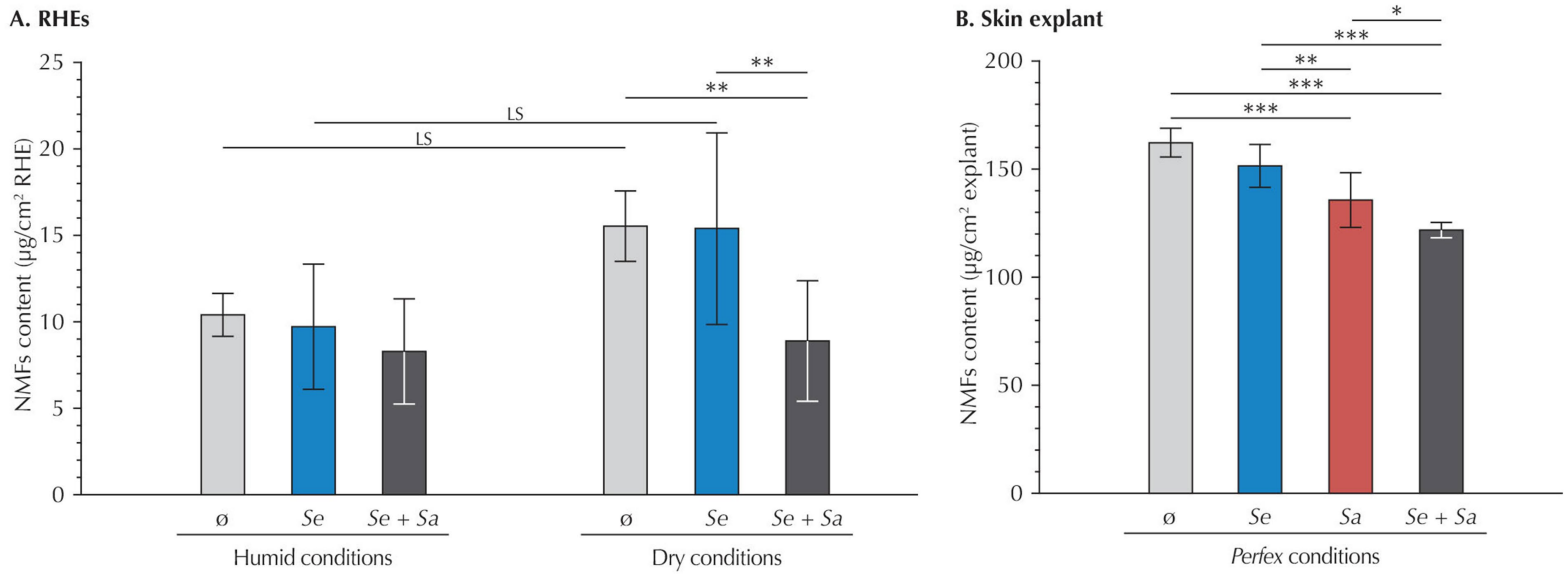
NMF content (serine, PCA and urocanic acid) in **(A)** RHEs and **(B)** *Perfex* skin explants. Results are expressed as mean ± standard deviation from measurements performed 24 h after inoculation on three different RHEs (each with duplicate technical repeats) or on three fragments of the same skin explant (each with triplicate technical repeats). Statistical differences are reported with LS: *p* < 0.1, *: *p* < 0.05, **: *p* < 0.01, and ***: *p* < 0.001.

To better understand the impact of bacterial colonization on skin hydration, we quantified three components from the NMFs – serine, pyrrolidone carboxylic acid (PCA), and urocanic acid (Figure 3).

In RHEs maintained under humid conditions, quantification of the three NMFs revealed no significant variation, regardless of whether RHEs were uninoculated or inoculated with *S. epidermidis*, *S. aureus*, or both. This absence of variation applied both to the total amount of the three NMF and to each individual compound (data not shown).

Under dry conditions, uninoculated RHEs showed a trend toward increased total NMF levels compared to those maintained under humid conditions (+54.7%, *p* = 0.092), although this difference was not statistically significant. At the individual compound level, increases remained non-significant or at the limit of significance (serine: *p* = 0.602; PCA: *p* = 0.100; urocanic acid: *p* = 0.116).

Besides, in RHEs subjected to dry conditions, inoculation with *S. epidermidis* alone did not alter total NMF levels compared to uninoculated controls (*p* = 1.000). However, co-inoculation with both bacterial strains significantly decreased the total content of the three NMFs (−42.7%, *p* = 0.025), restoring levels comparable to those observed under humid conditions (*p* = 1.000). This decrease was primarily driven by a reduction in PCA (−68.7%, *p* < 0.001), while changes in serine and urocanic acid levels were not statistically significant (−20.2%, *p* = 0.873 and −32.5%, *p* = 0.486, respectively).

Considering skin explants, inoculation with *S. epidermidis* alone had no significant impact on the total levels of the three NMFs compared to uninoculated samples (*p* = 0.110). In contrast, inoculation with *S. aureus* alone led to a 16% reduction in the total content of the three NMFs (*p* < 0.001), driven by significant decreases in all three components (−11% for serine, −19% for PCA, and −19% for urocanic acid; *p* < 0.001 for all). Co-inoculation produced an even greater reduction in the total levels of the three NMFs (−25%, *p* < 0.001), with consistent decreases across all components (−23% for serine, −18% for PCA, and −32% for urocanic acid; *p* < 0.001 for all). This reduction was also significant when compared to explants inoculated with *S. epidermidis* alone.

### 3.4 Assessment of the skin barrier function

**Figure 4:**
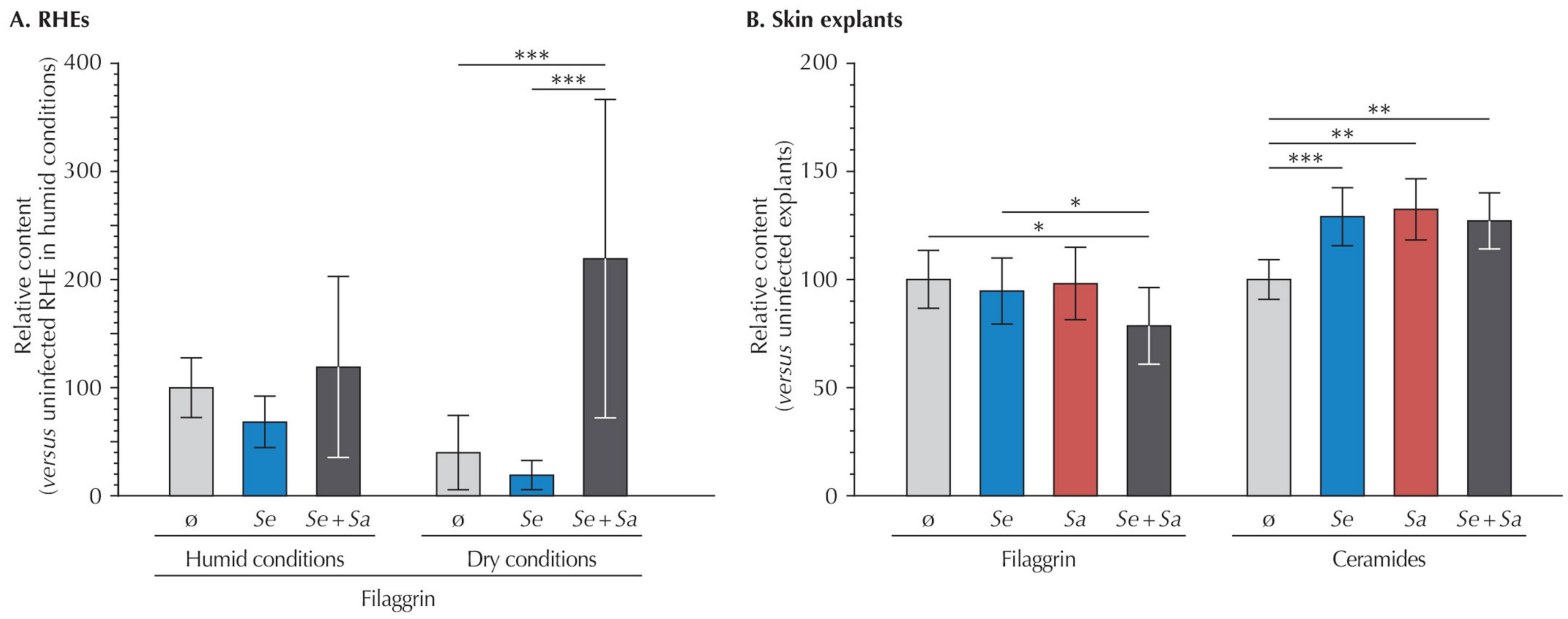
Relative quantification of filaggrin and ceramides in **(A)** RHEs (filaggrin only) and **(B)** Perfex skin explants, measured 24 h after bacterial inoculation. Results are expressed as mean ± standard deviation compared to the uninoculated control. For RHEs, quantifications were performed on three different RHEs (each with duplicate technical repeats). For skin explants, immunohistochemical labeling was quantified by analyzing at least three representative fields from cross-sections of three fragments of the same skin explant. Statistical differences are reported with *: *p* < 0.05, **: *p* < 0.01, and ***: *p* < 0.001.

To further investigate the effects of the microbiome model system on RHEs and skin explants, we next assessed skin barrier integrity by quantifying filaggrin (FLG) in RHEs and both FLG and ceramides in skin explants (Figure 4). As quantification methods differed between models, results were normalized to those of the respective uninoculated controls.

In RHEs maintained under humid conditions, bacterial inoculation by *S. epidermidis* or *S. epidermidis* and *S. aureus* had no significant effect on FLG expression compared to the uninoculated control. A comparison between uninoculated RHEs under humid and dry conditions revealed a non-significant decrease in FLG levels in the dry condition (*p* = 0.559). However, under dry conditions, co-inoculation with *S. epidermidis* and *S. aureus* significantly increased FLG expression, showing a 5.5-fold rise compared to uninoculated RHEs (*p* = 0.007), while inoculation by *S. epidermidis* had no impact (*p* = 0.996).

In contrast, skin explants exhibited a 21% decrease in FLG upon co-inoculation by *S. epidermidis* and *S. aureus* compared to uninoculated explants (*p* = 0.022), whereas bacteria alone had no effect (*p* = 0.328 for *S. epidermidis* and *p* = 0.783 for *S. aureus*).

Further investigating the barrier function of skin explants, we quantified ceramides, the most abundant lipid in corneocytes, the terminally differentiated cells forming the SC. Compared to uninoculated explants, inoculation by either bacterial strain alone or their co-inoculation resulted in higher ceramide levels (*S. epidermidis*: +29%, *p* < 0.001; *S. aureus*: +32%, *p* = 0.001, and +27%, *p* = 0.001 for the co-inoculation).

## 4 Discussion

Kohda et al. (2021) used RHEs to analyze the interaction of a skin commensal (*S. epidermidis*) and a pathogen (*S. aureus*). As generally accepted, they showed that 48 h after co-inoculation, *S. epidermidis* reduces *S. aureus* colonization compared to inoculation by *S. aureus* alone, without promoting its own growth. Performing a similar experiment, but focusing on an earlier time point, 4 h post-inoculation, we observed different results. Under the “classical” humid conditions, co-inoculation might enhance the adhesion of both *S. epidermidis* and *S. aureus*, an effect that is not significant when RHEs are maintained under dry conditions or in a more physiological model – basal-side-perfused skin explants maintained under standard environmental conditions. Furthermore, bacterial adhesion was approximately two orders of magnitude lower in skin explants than in RHEs.

Several factors could explain the discrepancies between our results and those of Kohda et al. (2021). A first consideration is the bacterial strains. Although we used different *S. epidermidis* strains for RHE and skin explant inoculations, the strain used in the skin explant experiments was the same as that employed by Kohda et al. (2021). Despite differences in origin and biofilm-related traits, the two strains are similar in the sense that they represent classical reference commensals and are generally regarded as presenting “balanced,” physiologically relevant models of beneficial and pathogenic skin colonization. A substantial difference lies in the *S. aureus* strains. The *S. aureus* subsp. *aureus* ATCC 6538™ strain we used is a slightly virulent but sensitive reference strain, representing a model of skin colonization and biofilm formation. By contrast, Kohda et al. (2021) used the ATCC BAA-1717™ strain, which is epidemic, highly virulent, and methicillin-resistant. It is generally considered as a robust pathological and infectious model. In addition to strain choice, the *S. epidermidis*-*S. aureus* inoculation ratio also differs between studies. This ratio is important, as the ability of *S. epidermidis* to limit *S. aureus* colonization is more effective when present in 100-fold excess compared to a 10-fold excess (Kohda et al., 2021). However, the outcome of a 1:1 co-inoculation ratio, as tested in our study, was not evaluated by Kohda et al. (2021).

Another important difference concerns the skin models. Although both studies used RHEs, they likely differed substantially. One key factor is the origin of the keratinocytes RHEs are derived from, neonatal foreskin *versus* adult skin in our case. This difference impacts RHEs’ architecture and the expression of various markers (Nedachi et al., 2023; Bajsert et al., 2024). Moreover, RHEs from both studies can only be from different donors which also affects their characteristics (Lange et al., 2016). Therefore, they could be significant variations in colonization dynamic as this dynamic vary between individuals (Kowalski et al., 2025). The lack of biological replicates in both studies – RHEs from multiple batches/donors and skin explants from a single donor in our case – further limits the generalizability and comparability of results. Finaly, important differences exist between RHEs and skin explants, particularly considering their barrier function properties (Schäfer-Korting et al., 2008; Nakamura et al., 2018), which further complicates direct comparisons across model systems.

While these limitations are important to consider, the difference observed between both studies could also arise from the different time points analyzed. Knowledges about the precise kinetic and the early events of skin colonization by both bacterial species remains limited. Skin colonization is preceded by an initial attachment phase, mediated by various bacterial components such as adhesins – among which the Accumulation-Associated Protein (Aap) in *S. epidermidis* and its ortholog, the *S. aureus* Surface protein G (SasG) in *S. aureus* – as well as various cell wall-anchored proteins binding the skin matrix – the largest family consisting of the Microbial Surface Components Recognizing Adhesive Matrix Molecules (MSCRAMMs), which are shared across both species (Paharik and Horswill, 2016; Josse et al., 2017; Severn and Horswill, 2023). The high degree of conservation of these adhesion molecules might explain the little to no differences observed, particularly given that many regulatory mechanisms active at later stages of colonization (24–48 h) are not yet functional at 4 hours. For instance, suppression of *S. aureus* by *S. epidermidis* through the quorum-sensing Accessory Gene Regulator (Agr) system depends on the accumulation of Auto-Inducing Peptideq (AIPs), which requires rather high bacterial densities (Le and Otto, 2015; Glatthardt et al., 2024), densities probably not reached at early time points. Similarly, the synthesis of antimicrobial peptides or the induction of host immune responses also requires more than 4 h to become effective (Pastar et al., 2020; Bier and Schittek, 2021). Biofilm formation and disruption, another key factor in bacterial persistence, is unlikely to be involved at this stage, as mature biofilms generally form after 24–48 h (Oliveira et al., 2007; Severn and Horswill, 2023).

Interestingly, under humid conditions (>90% relative humidity), *S. epidermidis* - *S. aureus* co-inoculation of RHEs led to enhanced bacterial attachment compared to single-species inoculation. This effect was not observed upon co-inoculation of RHEs maintained under dry conditions (<25% relative humidity) or in skin explants (40–60% relative humidity). The possible increased attachment observed upon co-inoculation suggests a potential synergistic interaction between the two bacterial species, specific to humid conditions. A direct effect of high humidity on bacteria could be possible as it positively influences bacterial metabolism and growth (Blanco et al., 2017; Qiu et al., 2022). It could also modulate host skin properties known to affect skin colonization (Bojar and Holland, 2002). However, these effects would be expected to apply equally to single and co-inoculated conditions, which is not the case. Still, *S. epidermidis* isolates from healthy skin modulate skin barrier function and immune responses via the Aryl hydrocarbon Receptor (AhR) pathway, but only under hydrated, conditions (Landemaine et al., 2023). In contrast, isolates from atopic dermatitis or dry skin lack these capacities, suggesting that a humid environment may be required for *S. epidermidis* to exert protective functions, support interspecies communication, and contribute to stable biofilm formation that could also include *S. aureus*.

Another notable observation is the 100-fold difference in bacterial attachment between RHE and skin explants. Although different methods were used to recover attached bacteria, quantification of unattached bacteria in explant cultures indicated that approximately half of the initial inoculum could be accounted for (data not shown), underscoring the lower attachment efficiency in this model. RHEs maintained in humid or dry conditions, are cultured at the air-liquid interface in microwell plates, allowing partial submersion and potential dynamic exposure to bacteria. In contrast, skin explants are positioned over a reservoir, exposing only their basal side to culture medium, with the stratum corneum exposed to the external environment. Inoculation in this context occurs under static conditions, resulting in less efficient bacterial deposition (Weidenmaier et al., 2018). Besides, as previously mentioned, the differences between the two models, particularly in their outermost layers, are substantial (Thakoersing et al., 2012). RHEs possess a non-desquamating stratum corneum with altered lipid content, reduced barrier function, and increased permeability. Skin explants, by contrast, maintain a fully differentiated stratum corneum, with native lipid organization and robust barrier properties. These features likely contribute to the lower bacterial adhesion observed in explants, with the reduced barrier function of RHEs possibly facilitating bacterial access to adhesion targets.

In contrast to the differential adhesion observed under humid conditions, changes in skin barrier components such as FLG and NMFs were primarily detected under dry conditions. Consistent with previous studies, dry conditions in uninoculated RHEs led to thickening of the stratum corneum and increased NMF levels (Cau et al., 2017), although the increase we observed was only at the limit of statistical significance. Besides, we also observed a non-significant decrease in FLG, potentially reflecting its degradation into NMFs.

Interestingly, only co-inoculation of RHEs maintained under dry conditions resulted in an increase in FLG levels and a concurrent reduction in NMFs, restoring values similar to those observed for RHEs maintained under humid conditions. Although *S. aureus*-only inoculations were not included in this series, skin explant results showed that *S. aureus* alone decreased NMF levels, with further reductions upon co-inoculation. Moreover, co-inoculated explants exhibited reduced FLG levels, unlike RHEs, suggesting opposite responses in the two models. Notably, both single and co-inoculation increased ceramide levels in explants.

All these results are difficult to fully explain. *S. epidermidis* has been reported to reinforce the skin barrier function by stimulating ceramide synthesis (Zheng et al., 2022), while *S. aureus* is known to impair epidermal barrier integrity and disrupt lipid metabolism (Nakatsuji et al., 2016; Kim et al., 2023). This makes the observed ceramide increase upon *S. aureus* inoculation somewhat unexpected. One possibility is that *S. aureus*-induced activation of keratinocyte stress pathways triggers compensatory upregulation of lipid synthesis, as part of a homeostatic response to barrier disruption (Kim et al., 2014). Alternatively, inflammatory cytokines induced by *S. aureus* (e.g., IL-1 family members, TNF-*α*) may indirectly stimulate ceramide production, as has been reported in epithelial stress models (Hänel et al., 2013; Feingold and Elias, 2024). Furthermore, *S. epidermidis* activation of AhR signaling may promote FLG synthesis (Rademacher et al., 2019; Furue, 2020), but studies on its impact on FLG expression remain inconsistent, with reports of no effect or decreased expression (Ochlich et al., 2023; Landemaine et al., 2023). In our experiments, single-species inoculation did not significantly affect FLG in either model. While our conditions were similar to those used by Ochlich et al. (2023), who observed decreased FLG in RHEs inoculated with *S. epidermidis*, our results showed only a non-significant reduction. This divergence might reflect context-dependent differences in AhR signaling outcomes, which can vary with ligand repertoire and local cytokine milieu (Denison and Faber, 2017). The situation is similarly complex for *S. aureus*. While it thrives on dry, barrier-compromised skin, its effect on FLG expression is poorly established (van Drongelen et al., 2014). Infection of isolated keratinocytes leads to increased protease activity and FLG degradation (Williams et al., 2017), suggesting that protease-mediated processing could underlie reductions in FLG. However, expression changes might be minimal, explaining our results in single-inoculation conditions. The only variation in FLG levels we observed were upon co-inoculation, with opposite results in RHEs and skin explants. While this opposite reaction may be due to the different bacterial strain used, it could also relate to the already discussed differences existing between the stratum corneum of RHEs and skin explants and the specificity of the effect of co-inoculation suggests a synergistic interaction between the two bacterial species. Potential mechanisms include competition for AhR ligands, convergence on cytokine signaling pathways, or altered protease activity resulting from interspecies crosstalk.

Notwithstanding the inconsistency in FLG expression, co-inoculation consistently led to decreased NMF levels in both RHEs and skin explants. Results from single-strain inoculations indicate that *S. aureus* contributes to this effect, and that co-inoculations exert a synergistic impact. Given that FLG is degraded into NMFs upon terminal differentiation of keratinocytes, the decrease in NMFs should have be accompanied by increased FLG, as was only evidenced in RHEs subjected to humid conditions. One hypothesis is that co-inoculation would trigger an immune reaction, with cytokine release reducing FLG expression, which might result in impaired barrier function and NMF loss. Alternatively, ceramide results could suggest that co-inoculation might improve the barrier function due to the stimulation of the *S. epidermidis* protective role by *S. aureus*, reducing the need for FLG synthesis and its degradation into NMFs.

Taken together, our study reveals unexpected interactions between *S. epidermidis*, *S. aureus*, and the skin. While co-inoculation may enhance bacterial attachment only under humid conditions, changes in skin barrier markers (FLG and NMFs) were observed only under dry conditions. Further studies are needed to confirm these findings and determine whether they are strain-dependent or not. Testing RHEs from different batches and various donors, as well as evaluating skin samples from various subjects will also be essential to assess whether results are generalizable. Investigating the early stages of bacterial colonization and their effects on skin biology will be essential, particularly in light of growing interest in the use of commensal bacteria as cosmetic agents or alternative therapies for skin diseases. These findings should also be considered in the broader context of skin diseases such as atopic dermatitis, in which *S. aureus* colonization is strongly shaped by the quorum-sensing mechanisms, antimicrobial peptide responses, and biofilm formation (Di Domenico et al., 2019; Okamoto et al., 2025). Assessing how the dysregulation of these pathways in AD not only facilitates *S. aureus* persistence but also amplifies inflammation, further highlights the clinical relevance of assessing the early events of host–microbe interactions.

## Data Availability

The original contributions presented in the study are included in the article/supplementary material, further inquiries can be directed to the corresponding author.
